# High-Throughput Sequencing Analysis of Small RNAs Derived from Coleus Blumei Viroids

**DOI:** 10.3390/v11070619

**Published:** 2019-07-05

**Authors:** Dong-Mei Jiang, Meng Wang, Shi-Fang Li, Zhi-Xiang Zhang

**Affiliations:** 1 Beijing Research Center for Agricultural Standards and Testing, Beijing Academy of Agriculture and Forestry Sciences, Beijing 100097, China; 2 State Key Laboratory of Biology of Plant Diseases and Insect Pests, Institute of Plant Protection, Chinese Academy of Agricultural Sciences, Beijing 100097, China

**Keywords:** viroid, small RNA, sRNA, coleus blumei, high-throughput sequencing

## Abstract

Characterization of viroid-derived small RNAs (vd-sRNAs) is important to understand viroid–host interactions; however, vd-sRNAs belonging to the genus *Coleviroid* are yet to be identified and characterized. Herein, we used coleus plants singly infected with coleus blumei viroid (CbVd)-1, -5, or -6 and doubly infected with CbVd-1 and -5 to identify and analyze their vd-sRNAs. We found sense and antisense vd-sRNAs for CbVd-1, -5 and -6, and 22-nt vd-sRNAs were the most abundant; moreover, the 5′-terminal nucleotides (nts) of CbVd-1, -5, and -6 were biased toward U and C, and sRNAs derived from these three viroids were unevenly distributed along their genomes. We also noted that CbVd-5 and -6 share a fragment that forms the right half of the rod-like secondary structure of these viroids, which implied that they generated almost the same type of vd-sRNAs. This finding indicated that vd-sRNA biogenesis is mainly determined by the primary sequence of their substrates. More importantly, we found two complementary vd-sRNAs (22 nt) that were generated from the central conserved region (CCR) of these three viroids, suggesting an important role of CCR in vd-sRNA biogenesis. In conclusion, our results provide novel insight into the biogenesis of vd-sRNAs and the biological roles of CCR.

## 1. Introduction

Coleus (*Coleus blumei* Benth.) is susceptible to six viroids: coleus blumei viroid (CbVd)-1 to -6 [1]. CbVd-1 to -3 have been recognized by the International Committee on Taxonomy of Viruses (ICTV) and assigned to the genus *Coleviroid*, which only contains these three species [2]; CbVd-4 to -6 [3,4,5] remain tentative species. Although no direct economic losses due to CbVd infections have yet been reported in coleus or other plant species, CbVds are good examples to illustrate the roles of recombination in the genetic variability and evolution of viroids and even RNA viruses [2] considering that they are prone to frequent genome-wide recombination. For instance, CbVd-2 and -6 are viroid chimeras—the former is made up of the right half of CbVd-1 and the left half of CbVd-3 [5] and the latter is made up of the right half of CbVd-5 and the left half of CbVd-3 [4]. Considering these distinctive features, studies on CbVds have not only been able to provide new insights into viroid evolution but also been helpful in understanding viroid biology. It was recently reported that a point-mutation in a loop in the secondary structure of CbVd-1 influences the seed transmission of CbVd-1 [6]. This significantly improved our understanding of the molecular mechanisms underlying the plant version of vertical transmission, and thus, more such studies are warranted.

In recent years, high-throughput sequencing (HTS), also referred to as next-generation or deep sequencing, has been widely applied in plant virology and has revolutionized both basic and applied research studies in this field, including but not limited to the discovery of novel viral agents, the detection and identification of known viruses and viroids, the analysis of genome diversity and evolution, and the study of the interactions between viral pathogens and plants [7,8]. Small RNA (sRNA) sequencing, a type of HTS technology, has been used to analyze and identify viroid-derived sRNAs (vd-sRNAs), which are known to be the hallmark of viroid-induced host RNA silencing, from members of the families of Pospiviroidae and Avsunviroidae [9,10,11,12,13,14]. Although these studies revealed the substrates for vd-sRNA biogenesis and characteristics of vd-sRNAs, the mechanism underlying vd-sRNA biogenesis still remains elusive.

HTS is now being widely used for studying plant pathogens considering that bioinformatic analysis is not only improving but also becoming cost effective [15,16]. HTS technologies such as sRNA and RNA sequencing (RNA-seq) have also been used for viroid identification [17,18,19,20] and discovery [21,22]; however, such methods are yet to be applied to study CbVds.

Herein, we used sRNA sequencing for the detection and identification of CbVd-1, -5, and -6 and the characterization of their vd-sRNAs. The complete genomes of CbVd-1, -5, and -6 could be assembled from their vd-sRNAs using different bioinformatic tools, helping in the establishment of methods to detect CbVds by HTS. vd-sRNA analyses of these viroids, including size distribution, 5’-terminal nucleotides (nts), and genome mapping, showed similar patterns and characteristics as those of other viroids, indicating that RNA silencing in the host is achieved using similar, overlapping mechanisms. More importantly, we could locate complementary vd-sRNAs derived from the central conserved region (CCR) of these three viroids. Collectively, the results reported in this study provide novel insight into the biogenesis of vd-sRNA and the biological roles of CCR.

## 2. Materials and Methods

### 2.1. Sample Preparation

In an earlier study [23], we obtained three coleus plants that were individually infected with CbVd-1, -5, and -6 through mechanical inoculations of dimeric CbVd (+) RNAs synthesized in vitro. The second or third fully opened young leaves from the top were collected three months post-inoculation and used for sRNA analysis by HTS. Coleus leaves from a field plant doubly infected with CbVd-1 and -5 were also collected for viroid detection.

### 2.2. RT-PCR and Northern Blot Hybridization

RT-PCR and northern blot hybridization were used to confirm the presence of CbVds. Total RNA was extracted using TRIzol (Tiangen Biotech, Beijing, China) according to the manufacturer’s instructions. cDNA was synthesized by M-MLV reverse transcriptase (Real-Times, Beijing, China) using random hexamer primers (Takara, Dalian, China), followed by PCR by Taq DNA polymerase using universal primers for the genus *Coleviroid* [24].

Northern blot hybridization was performed using a universal probe, as previously described [25]. Briefly, total RNAs were separated by 5% denaturing polyacrylamide gel electrophoresis and transferred onto a nylon membrane (Hybond-N+, Amersham Biosciences, Little Chalfont, UK). Hybridization was performed at 65 °C overnight using a universal cRNA probe for CbVds. Hybridization signal was generated using the chemiluminescent substrate CSPD (Roche, Basel, Switzerland) and chemiluminescence was detected by Bio-Rad ChemiDoc (Bio-Rad Laboratories, Hercules, CA, USA).

### 2.3. Cloning and Sequencing

The complete genomes of CbVd-1, -5, and -6 were amplified separately using pfu DNA polymerase (Takara, Dalian, China) with their specific primers, as previously described [3,4,26]. PCR products were gel purified, ligated into pTOPO vector (AidLab, Beijing, China) with blunt ends, and transformed into competent *Escherichia coli* cells (DH 5α). Positive clones were screened by RT-PCR and sequenced using 3730 XL DNA Analyzer (Thermo Fisher Scientific, Waltham, MA, USA).

### 2.4. Library Construction and sRNA Sequencing

Libraries for HTS were constructed with Illumina Small RNA Library Prep Kit (Invitrogen, waltham, MA, USA), according to the manufacturer’s instructions. Total RNAs that were extracted using TRIzol were employed as starting materials. The quality of the extracted RNA was confirmed by agarose gel electrophoresis, and the concentration was measured using a NanoDrop 2000 spectrophotometer (Thermo Fisher Scientific) and an Agilent 2100 Bioanalyzer (Agilent, Santa Clara, CA, USA). The workflow for constructing sRNA libraries included 3’- and 5’-terminal adapter ligation, RT-PCR, gel purification, size selection, qualification check, and library normalization; all protocols were performed as per the manufacturer’s instructions. The sRNA libraries were subsequently sequenced on an Illumina HiSeq 2000 (Illumina, San Diego, CA, USA) with the size of 2 × 100 bp.

### 2.5. Bioinformatic Analysis

Bioinformatic analysis was mainly performed for viroid identification and vd-sRNA characterization. After screening obtained raw reads for quality using Phred score, clean reads were produced by trimming adapter and low-quality reads by Trimmomatic v. 0.36 [27]. For viroid identification, the clean reads were assembled to contigs by Velvet v. 1.2.10 [28] and PFOR2 [21] using different k-mers of 15, 17, 19, and 21, and the resulting contigs were aligned with viral genomes [29] by BLAST [30]. For vd-sRNA characterization, the clean reads were mapped onto reference sequence(s) of CbVd-1, -5, and -6 for each sample using Bowtie 2 v. 2.3.4.3 [31] without mismatch.

## 3. Results

### 3.1. sRNA Sequencing and Quality Control

Coleus plants individually infected with CbVd-1, -5, and -6 [23] and a coleus leaf sample collected in the field were used for constructing sRNA libraries. Sequencing generated 16.55 M to 20.57 M raw reads (Table 1 and Table S1); trimming of adaptors and low-quality reads generated 15.95 M to 19.86 M clean reads (Table 1). Quality of the sequencing data was measured by the Q20 scores, length, GC content, and size distribution of the clean reads.

### 3.2. Detection of CbVd-1, -5, and -6 by sRNA Sequencing

sRNA sequencing, followed by bioinformatic analysis, has been widely used for the detection of viruses and viroids [15,16]. We accordingly used this technology in our study to detect CbVds. The obtained clean reads were de novo assembled using Velvet [28] and PFOR2 [21], followed by BLAST [29,30]. As expected, the contigs of CbVd-1, -5, and -6 were obtained from the individually infected coleus plants (Table 2), although the number, length, and genome coverage of viroid contigs assembled by Velvet and PFOR2 were obviously different. This observation for the individually infected coleus plants concurs with northern blot hybridization and RT-PCR results reported in an earlier study [23]. In addition, the contigs of CbVd-1 and -5 were obtained from the coleus leaf sample by both Velvet and PFOR2 (Table 2), also verified by RT-PCR and northern blot hybridization results, suggesting that sRNA sequencing can be used to detect CbVds, particularly CbVd-1 and -5, in the field condition.

### 3.3. Reference Sequences of CbVd-1, -5, and -6 for Read Mapping

**Figure 1:**
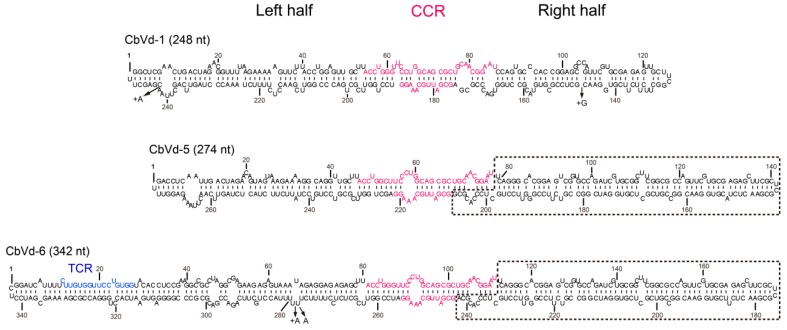
Predicted secondary structures of coleus blumei viroid (CbVd)-1, -5, and -6. The secondary structure of CbVd-1 refers to that determined by selective 2’-hydroxyl acylation analyzed by primer extension (SHAPE) [33]. The secondary structure of CbVd-5 and -6 was predicted based on their reference sequences, namely, FJ151371 and FJ615419, respectively. Different nucleotides between the reference sequence and infectious clone sequence are indicated by arrows. +A and +G means an A or G insertion in the corresponding position. The central conserved region (CCR) and terminal conserved region (TCR) are marked in pink and blue, respectively. Sequences shared by CbVd-5 and -6 are indicated by a dashed line.

The sequencing data obtained in the process of constructing the four sRNA libraries provided us with an opportunity to investigate the characteristics of sRNAs derived from CbVd-1, -5, and -6. Prior to this investigation, we determined the reference sequences of CbVd-1, -5, and -6. Viroid population includes a collection of closely related variants that are clustered around a master sequence; and this population structure has been described as “quasispecies” [32]. The master sequence is relatively stable and is a representative sequence variant in the population. It can thus be used as the reference sequence for screening vd-sRNAs from sequencing data. We accordingly used cloning and sequencing to determine the master sequences of CbVd-1, -5, and -6 in the four libraries. In the case of coleus plants singly infected with CbVd-1, -5, or -6, the master sequences of these three viroids were identical to the sequences of their infectious clones, based on previously reported results [23]. On the other hand, in the case of the leaf sample collected in the field, the master sequence of CbVd-1 was the same as that in the singly infected plant, whereas the master sequence of CbVd-5 was slightly different from that in the singly infected plant, that is, A was substituted by U at position 152 (Figure 1).

### 3.4. Characterization of sRNAs Derived from CbVd-1, -5, and -6

To characterize sRNAs derived from CbVd-1, -5, and -6, the sequencing data of the four sRNA libraries were mapped onto the corresponding reference sequences that were previously determined without mismatch to obtain 21–24-nt long vd-sRNAs. We found that the total percentage of vd-sRNA was 2.83–5.74% of the clean reads in the sRNA libraries (Table 1). Notably, although the number of clean reads (17.53 M) in the case of coleus plants doubly infected with CbVd-1 and -5 was a little less than that in those singly infected with CbVd-1 or -5 (19.41 M and 19.86 M, respectively), the number of CbVd-1 and -5 vd-sRNAs in doubly infected plants was approximately twice as high as that in singly infected plants. This indicated that CbVd-1 and -5 generated more vd-sRNAs in the presence of a co-infection, which may be explained by the long-term infection and higher accumulation level of these two viroids in doubly infected coleus plants collected in the field.

#### 3.4.1. Polarity

**Figure 2:**
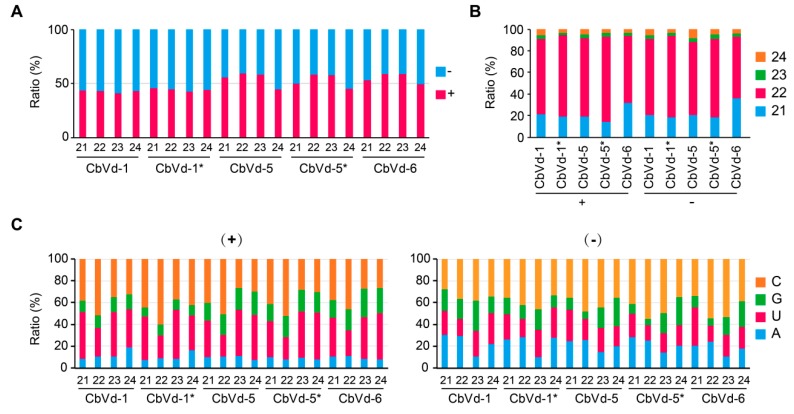
(**A**) Polarity, (**B**) size, and (**C**) 5’-terminal nucleotides of sRNAs derived from CbVd-1, -5, and -6. Asterisk indicates that the viroid was from coleus plants in the field doubly infected with CbVd-1 and -5.

Both sense and antisense vd-sRNAs were found for CbVd-1, -5, or -6 in the four libraries (Table 1, Figure 2A), indicating the involvement of viroid replication intermediates, antisense genomic or double-stranded RNAs, in vd-sRNA biogenesis. The ratios of sense and antisense vd-sRNAs seemed to be determined by the type of viroid. The ratio was <1 for CbVd-1 in both singly (0.75) and doubly (0.81) infected plants but >1 for CbVd-5 in both singly (1.35) and doubly (1.28) infected plants and for CbVd-6 (1.29) in singly infected plants (Table 1). Similar results were observed for sRNA size, which ranged from 21 to 24 nt (Figure 2A). On the basis of these observations, we concluded that vd-sRNAs of CbVd-1, -5, and -6 are produced from both their genomic and complementary RNAs, but in disproportionate quantities.

#### 3.4.2. Size Distribution

In line with previous observations that the majority of vd-sRNAs are 21 and 22 nt in length in the case of viroids belonging to the family Avsunviroidae and Pospiviroidae [34], 21- and 22-nt long vd-sRNAs were also predominant in all the sRNA libraries for both sense and antisense polarities (Figure 2B). However, 22-nt vd-sRNAs were much more abundant (>2-fold) than 21-nt vd-sRNAs in CbVd-1-, -5-, or -6-infected plants. In contrast, in hop stunt viroid (HSVd)- and grapevine yellow speckle viroid-infected grapevine, HSVd-infected cucumber leaf, peach latent mosaic viroid-infected peach trees, and apple hammerhead viroid-infected apples, the most abundant size class of vd-sRNAs was 21 nt. This result indicates that in coleus, dicer-like enzyme-2 (DCL2) plays a major role in the biogenesis of sRNA of CbVd-1, -5, and -6. In addition to 21- and 22-nt vd-sRNAs, accumulation of 24-nt vd-sRNAs [10,34,35] was found in all the sRNA libraries for both sense and antisense polarities (Figure 2B); however, the abundance of 24-nt vd-sRNAs was as low as that of 23-nt vd-sRNAs.

#### 3.4.3. 5’-terminal nucleotide

The sorting process of sRNAs into effector Argonaute (AGO) proteins is mainly determined by their 5’-terminal nucleotide [36]. We believe that vd-sRNA sorting also follows this principle as it has been reported that the sorting process of sRNAs of potato spindle tuber viroid into AGO1, AGO2, AGO4, and AGO5 is largely conditioned by the 5’-terminal nts of sRNAs [37]. Thus, we analyzed the 5’-terminal nts for each size of sRNAs derived from CbVd-1, -5, and -6 in both sense and antisense polarities (Figure 2C). In the sense polarity, C and U were prevalent, particularly in the case of 21- and 22-nt vd-sRNAs, whereas in the antisense polarity, C was prevalent, followed by U and A, and the frequency of U and A showed no obvious differences. Therefore, similar to other viroids belonging to the families Avsunviroidae and Pospiviroidae, the 5’-terminal nts of CbVd-1, -5, and -6 appeared to be biased toward C and U.

### 3.5. Distribution of sRNAs Derived from CbVd-1, -5, and -6 along Their Genomes

**Figure 3:**
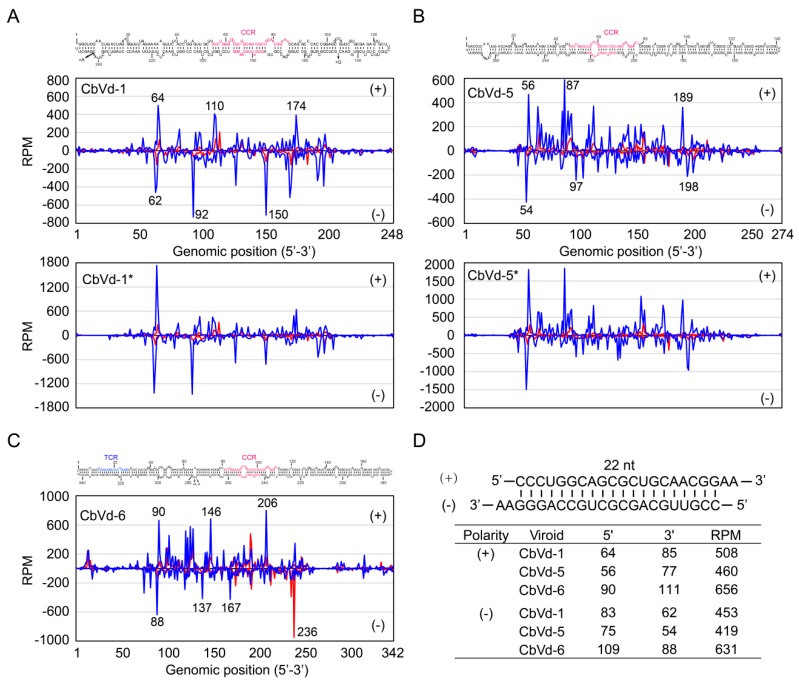
Distribution of sRNAs derived from (**A**) CbVd-1, (**B**) -5, and (**C**) -6 along their genomes. sRNA of 21 nt is indicated in red and that of 22 nt in blue. The location of sRNAs indicated the position of 5’-terminal nucleotides of (+)-sRNA and that of 3’-terminal nucleotides of (−)-sRNA. Locations of some hotspots are specifically indicated. An asterisk indicates that the viroid was from coleus plants in the field doubly infected with CbVd-1 and -5. (**D**) Sequences, location, and read numbers of two complementary vd-sRNA (22 nt) from CbVd-1, -5 and -6.

To reveal the distribution of sRNAs derived from CbVd-1, -5, and -6 on the genomes of these viroids, mapping of vd-sRNAs onto the reference genome was performed using Bowtie 2 [31] without mismatch. The location in the genome and the abundance of 21- and 22-nt vd-sRNAs were illustrated for CbVd-1, -5, and -6 for both singly and doubly infected coleus plants (Figure 3A–C). Here, the location of vd-sRNAs in the genome indicates the position of 5’-terminal nts of sense vd-sRNAs and that of 3’-terminal nts of antisense vd-sRNAs. We observed that sRNAs derived from CbVd-1, -5, and -6 were unevenly distributed along their genomes with some hotspots (Figure 3A–C). The sRNA distribution pattern of CbVd-1, -5, and -6 was similar to that previously reported for some other viroids [9,10]. It should be noted that vd-sRNA distribution in the genomes of CbVd-1 and -5 was very similar between singly and doubly infected coleus plants (Figure 3A,B).

We also found that vd-sRNAs were mainly distributed in the middle of the genome of CbVd-1, -5, and -6, but rarely in the beginning or end of the genomes (Figure 3A–C). In other words, the majority of vd-sRNAs were derived from the right halves of the predicted rod-like secondary structures of CbVd-1, -5, and -6. In contrast, only a few vd-sRNAs were derived from the left halves, particularly from the terminal left regions, of the predicted rod-like secondary structures of CbVd-1, -5, and -6. These results indicate that right halves of the secondary structures of these three viroids are more susceptible to cleavage by coleus DCLs, and they thus have higher selective pressure mediated by RNA silencing.

Upon closely inspecting the locations of some hotspots of vd-sRNA distribution in the genome, we found at least one base-paired vd-sRNA of the hotspots each for CbVd-1, -5, and -6 (Figure 3A–C). It is noteworthy that double-stranded sRNAs show a two-nt overhang at the 3’-end. Thus, the vd-sRNA of the hotspot at position 64 in the sense and that of the hotspot at position 62 in the antisense genome of CbVd-1 were base-paired (Figure 3A). Similarly, the vd-sRNAs of the hotspots at positions 56 and 54 in CbVd-5 (Figure 3B) and those of the hotspots at positions 90 and 88 in CbVd-6 (Figure 3C) were also base-paired. Strikingly, although these three base-paired sRNAs were respectively derived from CbVd-1, -5, and -6, their sequences were the same, as they are derived from CCR, a highly conserved structural motif among different viroids [2], of the predicted secondary structures of these three viroids (Figure 3D). This observation suggests that CCR, as a substrate, is susceptible to cleavage by coleus DCLs. It is worth noting that CCR and its flanking nts form a conserved double-stranded structure or hairpin I that is the substrate for in vivo cleavage during viroid replication [38]. This suggests that a possible link exists between vd-sRNA biogenesis and viroid replication.

### 3.6. vd-sRNA Distribution on the Shuttling Genomic Fragment between CbVd-5 and -6

**Figure 4:**
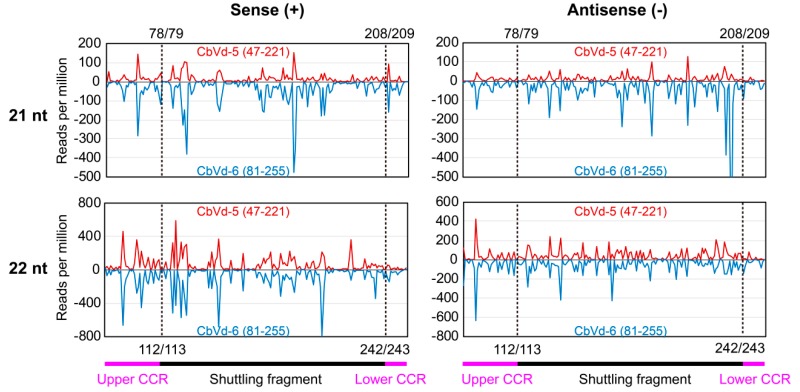
Distribution of sRNAs (21 and 22 nt) on the genomic fragment shared by CbVd-5 and -6. Sense strand is indicated in red and antisense strand in blue. Locations of CCR and shuttling fragment on CbVd-5 and -6 genomes are 47–221 and 81–255, respectively. A dashed line indicates the boundary between shuttling fragment and CCR (upper and lower).

As CbVd-6 is a viroid chimera made up of the left half of CbVd-3 and the right half of CbVd-5 based on the predicted rod-like secondary structure [4], CbVd-5 and -6 share a fragment that forms the right half of their rod-like secondary structure (Figure 1). The presence of a shuttling genomic fragment between CbVd-5 and -6 provided us with an opportunity to investigate whether a genomic fragment shared by different viroids can generate similar sRNAs. We thus investigated vd-sRNA distribution on the genomic fragment shared by CbVd-5 and -6, spanning positions 47–221 in CbVd-5 and 81–255 in -6 (Figure 4). Although the abundance of CbVd-6-sRNAs was a little higher than that of CbVd-5-sRNAs, vd-sRNA distribution patterns on the shared genomic fragment were almost the same in both sense and antisense polarities (Figure 4). In addition, analyses of unique vd-sRNAs showed that the genomic fragment shared by CbVd-5 and -6 generated almost the same type of vd-sRNAs. These results indicated that genomic sequences seem to be the main determinant for vd-sRNA biogenesis.

## 4. Discussion

Viroid infection induces RNA silencing in the host plant with the accumulation of vd-sRNAs, which are usually used for viroid detection and identification and for studying viroid–host interactions through HTS [10,15,18,33]. Here, sRNAs derived from coleus plants singly or doubly infected with CbVd-1, -5, and -6 were analyzed by sRNA sequencing. Using the obtained data, we assembled the complete genomes of these viroids and identified the size, polarity, 5’-terminal nt, and distribution of sRNAs derived from CbVd-1, -5, and -6 along the genome of these three viroids. Thus, a novel method for detecting CbVds was developed, which helped us elucidate the characteristics of vd-sRNAs belonging to the genus *Coleviroid*, providing several insights into the interaction between CbVds and coleus plants.

Full-length genome of viroids can be assembled using sRNA sequencing data as vd-sRNAs span the entire viroid genome and also overlap each other [18,39]. In this study, the contigs of CbVd-1, -5 and -6 were obtained from individually infected coleus plants and then assembled de novo by PFOR2, a computational algorithm developed for assembling circular RNA [21]. However, a chimera sequence, containing the right and left halves of CbVd-1 and -5, respectively, was also assembled by PFOR2. The reason for this false assembly could be the CCR shared by CbVd-1 and -5. It is speculated that shuttling genomic fragments between viroids of the genus *Coleviroid* can result in the generation of ambiguous results for viroid detection and identification, particularly if a plant is co-infected with at least two CbVds. Thus, RT-PCR should be used to verify viroid sequences assembled using sRNA sequencing.

In comparison with other viroids, CbVd-1, -5, and -6 showed a higher abundance of 22-nt vd-sRNAs in all the sRNA libraries for both sense and antisense polarities, implying that coleus DCL2 plays a key role in vd-sRNA production. Given that DCL-mediated cleavage of double-stranded viroid replication intermediates or double-stranded RNAs generated by host RNA-directed RNA polymerases (RDRs) is one of the barriers for defense against viroid infection [40], it is speculated that coleus DCL2 may be critical for antiviroid defense. It was recently reported that the combined suppression of DCL2 and DCL3 has a major effect in succumbing plant antiviroid defense in a series of DCL-knockdown plants of *Nicotiana benthamiana* [41,42]. The role of coleus DCL2 in antiviroid defense should thus be experimentally verified in future studies.

Although the origin of vd-sRNAs remains elusive, it is known that viroid-related double-stranded RNAs, mainly including viroid replication intermediates and double-stranded RNAs synthesized by host RDRs, are substrates of DCL cleavage as both sense and antisense vd-sRNAs have been found in viroid-infected hosts. In principle, there should be a lot of complementary vd-sRNAs with a two-nt overhang at the 3’-end in viroid-infected hosts. However, complementary vd-sRNAs were not common in previous studies [9,10,11,12,13,14]. In the present study, we observed two complementary vd-sRNAs (22 nt) in coleus plants singly or doubly infected with CbVd-1, -3, and -5, and they were derived from the upper strand of CCR in the predicted secondary structure (Figure 1) and its complementary sequence, respectively. Importantly, they are two hotspots of vd-sRNAs in these three viroids. These findings indicate that CCR of these three viroids could be recognized and cleaved by coleus DCL2.

CCR plays a critical role in viroid replication, being involved in both cleavage and ligation through two structural motifs of the conserved double-stranded structure and loop E [38]. Upon entering host cells, viroids initiate replication and accumulate high levels of genomic RNAs. In this process, host RNA silencing is induced, which limits the accumulation of viroid genome through cleavage of viroid-related double-stranded RNAs and genomic RNAs by host DCLs and AGOs, respectively. Viroid replication and antiviroid defense are counteracted and both are associated with CCR. The interaction between these two counteracted processes should reach a dynamic balance, which is evidently regulated by the conserved double-stranded structure and loop E in CCR and perhaps also by complementary vd-sRNAs in CCR. Thus, CCR, at least in the genus *Coleviroid*, may have more biological functions than previously reported.

CbVd-5 and -6 share a fragment that forms the right half of the rod-like secondary structure of these viroids [3,4]. This gave us an opportunity to investigate whether a genomic fragment shared by various viroids could generate similar vd-sRNAs. Our results showed that the genomic fragment shared by CbVd-5 and -6 indeed produced almost the same vd-sRNA distribution of vd-sRNAs; this result is in line with the observation that profiles of virus-derived sRNAs were very similar to those obtained for the transgene in the relevant region [43]. Thus, genomic sequences are perhaps the main determinant for vd-sRNA biogenesis.

In summary, we comprehensively analyzed sRNAs derived from CbVd-1, -5, and -6 using singly and doubly infected coleus plants by sRNA sequencing and also investigated their characteristics including size, polarity, 5’-teminal nts, and distribution on genome. More importantly, the presence of complementary vd-sRNAs in CCR of these three viroids indicates a more important biological role of CCR in vd-sRNA biogenesis as well as in replication.

## Figures and Tables

**Table 1:** Number of sequencing reads of coleus blumei viroid (CbVd)-1-, -5-, and -6-derived small RNAs (sRNAs).

Viroid	Total Reads (Million)	Clean Reads (Million)	vd-sRNAs ^#^		
			Total (%)	+ (%)	− (%)
CbVd-1 *	18.53	17.53	700,128 (3.994)	312,876 (1.785)	387,252 (2.209)
CbVd-1	20.11	19.41	568,154 (2.927)	243,169 (1.253)	324,985 (1.674)
CbVd-5 *	18.53	17.53	1,006,161 (5.74)	566,058 (3.229)	440,103 (2.511)
CbVd-5	20.57	19.86	562,821 (2.834)	323,465 (1.629)	239,356 (1.205)
CbVd-6	16.55	15.95	789,359 (4.949)	444,061 (2.784)	345,298 (2.165)

* from coleus plant doubly infected with CbVd-1 and -5; ^#^ viroid-derived small RNAs of 21–24 nt.

**Table 2:** Viroid contigs obtained using Velvet and PFOR2.

Viroid	Velvet			PFOR2		
	Number	Length (nt)	Coverage	Number	Length (nt)	Coverage
CbVd-1	28	31, 33	71.4%	4	247–250	100%
CbVd-5	42	33, 53	64.2%	2	274	100%
CbVd-6	38	31, 33	75.4%	3	341–342	100%
CbVd-1 *	9	33	66.7%	1	247	100%
CbVd-5 *	4	33	48.2%	1	274	100%

* from coleus plant doubly infected with CbVd-1 and -5.

## Supplementary Materials

The following are available online at https://www.mdpi.com/1999-4915/11/7/619/s1, Table S1: Quality control of sRNA sequencing data.
