# Supplementary material for: High-Throughput Sequencing Analysis of Small RNAs Derived from Coleus Blumei Viroids
Source: Viruses. 2019 Jul 5;11(7):619. doi: 10.3390/v11070619 (PMC6669434; doi:10.3390/v11070619)
Supplement: Supplementary file 1 [file viruses-11-00619-s001.pdf]

Supporting information

Table S1. Quality control of sRNA sequencing data

Sample	Raw reads (M)	Minimum length (nt)	Maximum length (nt)	Average length (nt)	Q20 (%)	G/C content (%)
CbVd-1	20.11	10	50	22.47	99.58	46.67
CbVd-5	20.57	10	50	22.35	99.59	47.70
CbVd-6	16.55	10	50	22.39	99.58	47.91
CbVds *	18.57	10	50	22.74	99.65	48.98

* from coleus plant doubly infected with CbVd-1 and -5.
